# Optimal antiplatelet therapy in patients with acute coronary syndrome and chronic kidney disease: for the Jerusalem platelets thrombosis and intervention in cardiology (JUPITER-11) study group

**DOI:** 10.1186/s12882-025-04227-8

**Published:** 2025-08-04

**Authors:** Perel Nimrod, Marmor David, Taha Louay, Tabi Meir, Itshak Amsalem, Rafi Hitter, Tomer Maller, Nir Levy, Lior Lupu, Hezzy Shmueli, Sharon Bruoha, Tal Ovdat, Michael Glikson, Elad Asher

**Affiliations:** 1 Jesselson Integrated Heart Center, Shaare Zedek Medical Center and Faculty of Medicine, Hebrew University of Jerusalem, Jerusalem, Israel; 2 Department of Cardiology, Tel Aviv Medical Center, Affiliated to the Sackler Faculty of Medicine, Tel Aviv University, Tel Aviv, Israel; 3 Department of Cardiology, Soroka Medical Center, Beer-Sheva, Israel and Faculty of Health Sciences, Ben-Gurion University of the Negev, Beer-Sheva, Israel; 4 Department of Cardiology, Barzilai Medical Center, The Ben-Gurion University of the Negev, Beer-Sheva, 8410501 Israel; 5 The Israeli Center for Cardiovascular Research, Ramat Gan, Israel

**Keywords:** Antiplateletu therapy, Acute coronary syndrome, Chronic kidney disease

## Abstract

**Introduction:**

Chronic kidney disease (CKD) is a well-documented risk factor for major adverse cardiac events and bleeding events. The optimal antiplatelet strategy for patients with CKD remains unclear, especially patients with glomerular filtration rate (GFR) < 30 ml/min. We aim to compare clinical outcomes of patients with acute coronary syndrome (ACS) and CKD treated with ticagrelor or prasugrel vs. clopidogrel.

**Methods:**

Patients were collected from the acute coronary syndrome Israeli survey (ACSIS). Patients were divided into 2 groups: ST-segment myocardial infarction (STEMI) and non-STEMI. Each group was further divided based on the GFR value (<30 ml/min or ≥ 30 ml/min). Mortality, bleeding, repeat revascularization, and re-hospitalization at 30-day and 1-year were evaluated.

**Results:**

A total of 5,442 patients were included in the final analysis. There were no significant differences regarding baseline characteristics between both groups of ACS patients. In patients with STEMI and GFR < 30 ml/min, re-hospitalization (32% vs. 29 % p = 1.0), bleeding (2.7% vs 2.3% p = 0.9), and death rates (8.1 % vs 2.3% p = 0.5) did not differ between the two different treatment strategies at 30-day follow-up. The mortality rate in 1-year was lower among STEMI patients treated with the ticagrelor or prasugrel as compared with clopidogrel (33% vs 11% p = 0.04).

**Conclusion:**

In patients with ACS and CKD, rehospitalization, bleeding and mortality rates were similar in30 days in patients treated with ticagrelor/prasugrel versus clopidogrel. Nevertheless, in patients presenting with STEMI and GFR < 30 ml/min, treatment with ticagrelor/prasugrel was correlated with a lower 1-year mortality rate as compared with clopidogrel treatment with no increase in bleeding rates.

## 1. What is known about this topic

•Potent antiplatelet therapy is the current standard of treatment for patients with acute coronary syndrome (ACS), however the optimal antiplatelet strategy for patients with chronic kidney diseaseany difference in the sub analysis for either remains unclear, especially patients with glomerular filtration rate (GFR) < 30 ml/min

## 2. What does this paper adds

In patients with advanced CKD and STEMI treatment with potent antiplatelet regimen was associated with a lower 1 year mortality rate.

Our finding, including mortality, ischemic and bleeding event,adds additional information about the optimal antiplatelet therapy in patients with advanced CKD diagnosed with ACS.

## Introduction

Chronic kidney (CKD) disease is a common comorbidity in patients with acute coronary syndrome (ACS). Reduction of renal function in patients with any type of ACS is associated with worse outcomes [1, 2].

Patients with CKD have a higher rate of both ischemic events and bleeding. The mechanism of a higher thrombotic risk results from endothelial injury disrupting the normal antithrombotic function of the healthy endothelium, while altered aggregation and adhesion contribute to increased bleeding risk. This double edge sword represents a constant challenge when choosing an antiplatelet therapy in these patients [3, 4].

Two previous major antiplatelet trials, the PLATO (Platelet Inhibition and Patient Outcomes) and the TRITON -TIMI 38 (Trial to Assess Improvement in Therapeutic Outcomes by Optimizing Platelet Inhibition With Prasugrel–Thrombolysis In Myocardial Infarction 38), showed significant benefits of ticagrelor and prasugrel therapy in ACS patients [5, 6]. Subgroup analyses of CKD patients in these trials showed benefit from prasugrel, but not ticagrelor [5, 7]. A subgroup analysis of the ISAR-REACT 5 trial (Intracoronary Stenting and Antithrombotic Regimen: Rapid Early Action for Coronary Treatment) that examined patients with low GFR, showed no outcomes differences between prasugrel and ticagrelor in patients with GFR < 60 ml/min when compared to the overall population [8, 9]. Current guidelines indicate that the choice and dose of antithrombotic drugs should be carefully considered in patients with CKD, with no clear recommendation on which type of P2Y12 inhibitors should be used in this high risk population [10, 11].

Hence, our study aimed to investigate the safety and efficacy of the different antiplatelet therapies in patients with ACS and CKD in a real-world setting.

## Methods

### Study population

This study included consecutive patients from the ACS Israeli Surveys (ACSIS) between 2013 and 2021.The ACSIS registry has been described previously [12], in brief, it is a prospective survey conducted every 2–3 years, which includes patients from all 26 intensive cardiac care units (ICCU’s) operating in Israel over a consecutive 2-month period. The data are entered electronically by dedicated and specifically trained research personnel. The pre-specified demographic, CV risk factors, co-morbidities, medications and clinical data are recorded along with admission and discharge diagnosis as defined by the attending physicians based on clinical, electrocardiographic, and biochemical criteria. Informed consent is obtained on all patients. The institutional review board (IRB) of all the participating hospitals approved the survey, which was performed in accordance with the Helsinki declaration.

### Cohort of the study

Patients with ST elevation myocardial infarction (STEMI), non-ST segment myocardial infarction (NSTEMI), and unstable angina pectoris (UAP) who survived the index hospitalization with available glomerular filtration rate (GFR) (mL/min/1.73 m²) values, and available data of the antiplatelet treatment regimen at time of discharge were included in the study.

Patients were then divided into STEMI and NSTEMI/UAP groups. Each group was further divided into GFR < 30 ml/min and GFR ≥ 30 ml/min and was stratified for the different P2Y12 antiplatelet regimens (clopidogrel vs ticagrelor or prasugrel). The decision to set the eGFR cutoff at 30 rather than the more conventional 60 is rooted in the study’s aim to focus on a subset of patients with more severely impaired renal function.

### Outcomes

We evaluated patients’ outcomes at 30 days and at 1 year for mortality. We evaluated rehospitalization, recurrent myocardial infarction (MI), and major and minor bleeding outcome at 30 days. Bleeding events were categorized according to the TIMI (Thrombolysis in Myocardial Infarction) criteria. Major bleeding was defined as any intracranial hemorrhage or any clinically overt signs of hemorrhage associated with a drop in hemoglobin of ≥ 5 g/dL or a fatal outcome. Minor bleeding was defined as any clinically overt sign of hemorrhage resulting in a drop in hemoglobin of 3 to < 5 g/dL [13].

Recurrent myocardial infarction (MI) was defined by the presence of new symptoms or signs consistent with myocardial ischemia, accompanied by new electrocardiographic changes indicative of new ischemia (new– changes or new left bundle branch block), or by the elevation of cardiac biomarkers to at least twice the upper limit of normal [14].

In addition, the combined outcome of recurrent ischemic event with or without bleeding was calculated.

### Statistical methods

Patient characteristics were presented as n (%) for categorical variables, and as mean (sd) or median (IQR) for normal/non-normal distributed continuous variables. The study-groups were tested with a chi-square test for categorical variables and with t-test or Mann–Whitney–Wilcoxon test as appropriate for normal/nonnormal distributed continuous variables.

Survival curves were plotted, and the Kaplan-Meier log rank test was used to test the medication (ticagrelor/prasugrel versus clopidogrel) on the outcome 1-year mortality, among the different diagnoses and GFR groups.

In order to assess the relationship between medication and outcomes among the different diagnoses and GFR groups, a logistic regression model for the combined outcome in 30 days, and a Cox proportional hazards regression model for the outcome of 1-year mortality, were performed, adjusted for pre specified covariates: age, gender, and ejection fraction. Interaction terms between GFR groups and medications were assess.

The propensity score evaluates the probability for GFR < 30, using a logistic regression model. In STEMI patients, the following covariates were selected: age, gender, hypertension, prior CHF. PSM was performed with a caliper of 0.01 and a 1:2 matching (low:high GFR).In NSTEMI/UAP patients, the following covariates were selected: age, gender, hypertension, dyslipidemia, diabetes, prior PVD, prior CHF, GRACE score > 140. PSM was performed with a caliper of 0.02 and a 1:2 matching (low:high GFR).

**Fig. 1:**
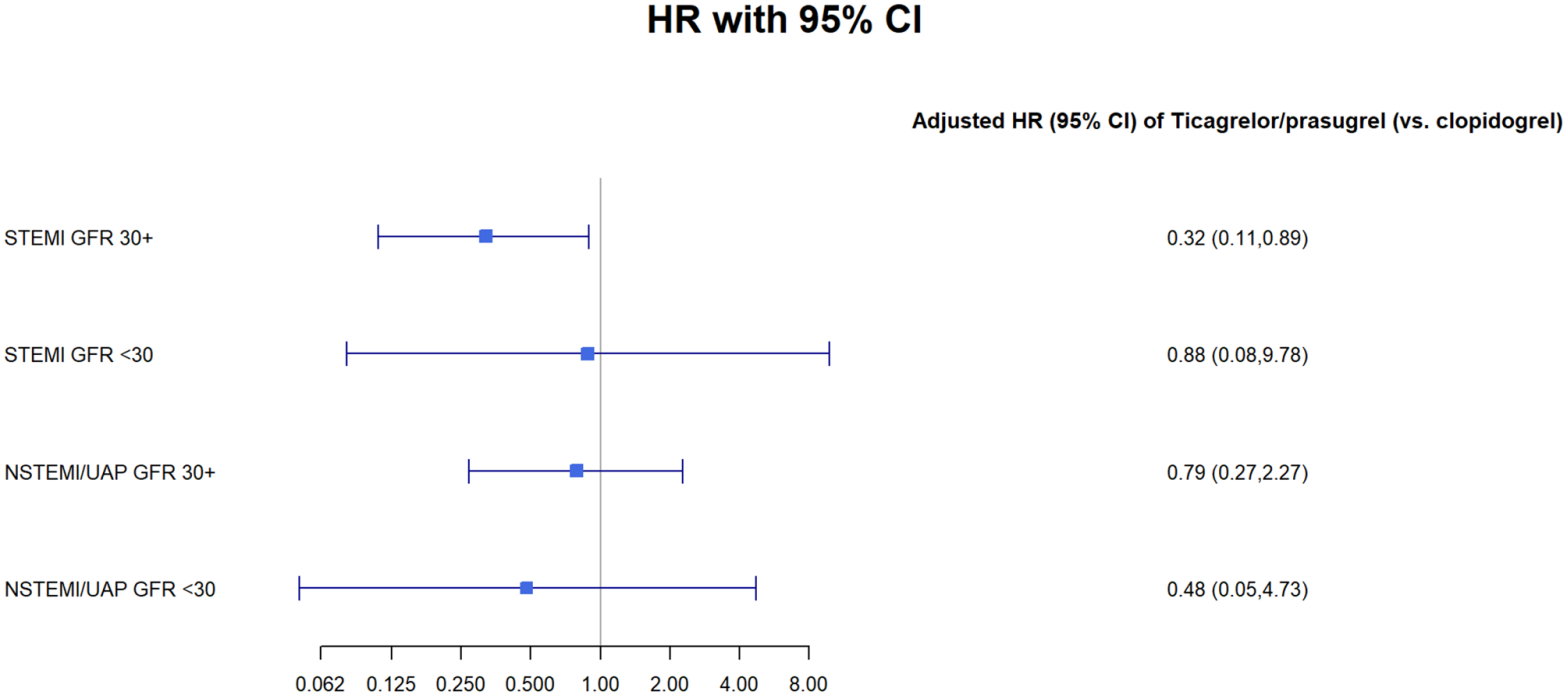
GFR- glomerular filtration rate; STEMI- ST elevation myocardial infraction; NSTEMI- non-ST elevation myocardial infraction; UAP- unstable angina pectoris

**Fig. 2:**
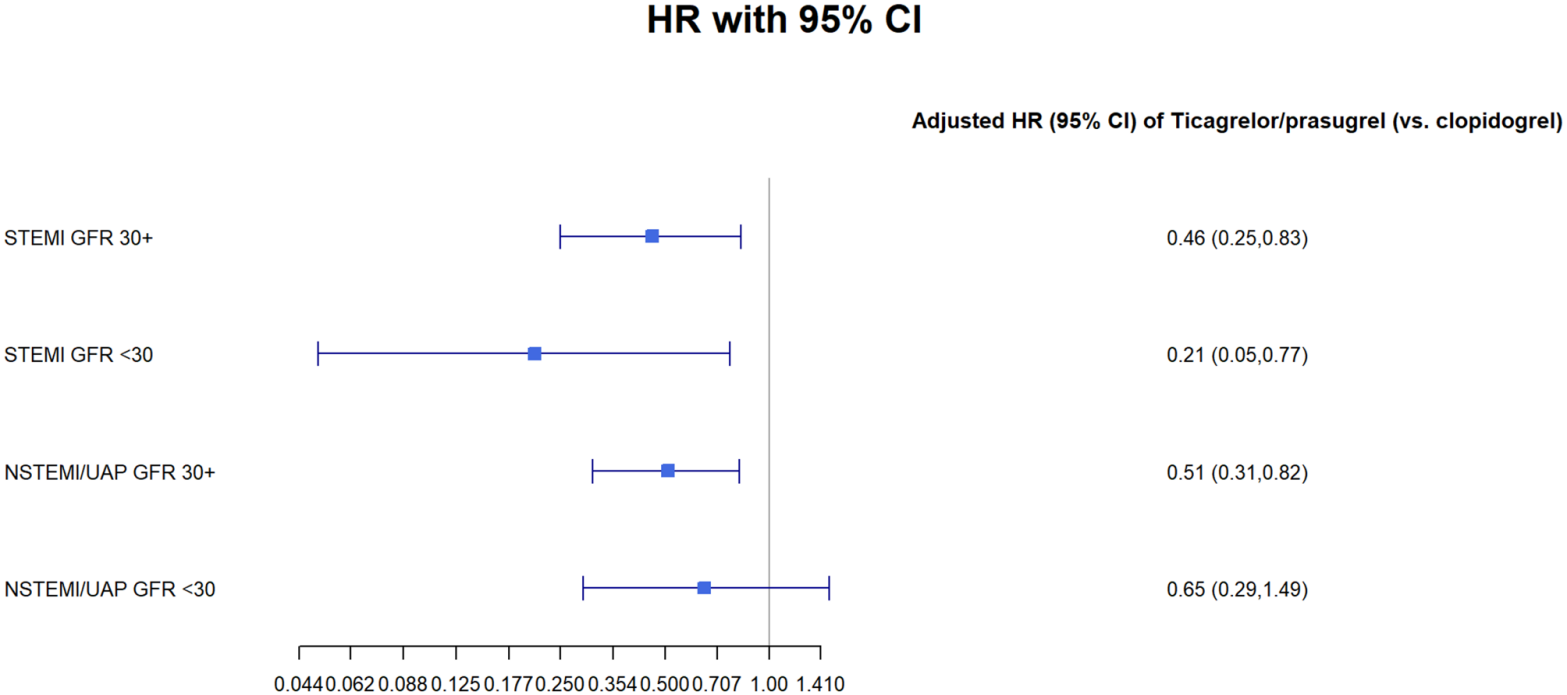
GFR- glomerular filtration rate; STEMI- ST elevation myocardial infraction; NSTEMI- non-ST elevation myocardial infraction

**Fig. 3:**
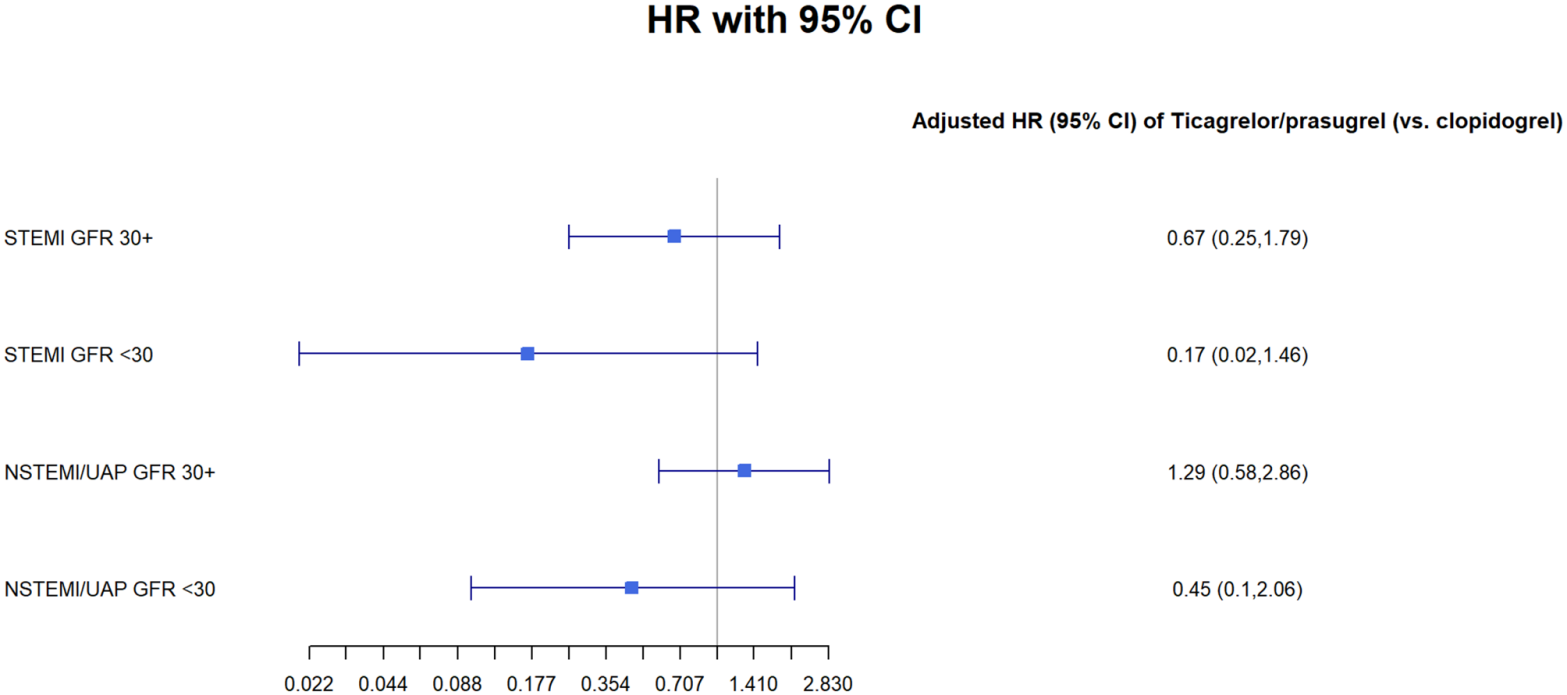
GFR- glomerular filtration rate; STEMI- ST elevation myocardial infraction; NSTEMI- non-ST elevation myocardial infraction; UAP- unstable angina pectoris

**Fig. 4:**
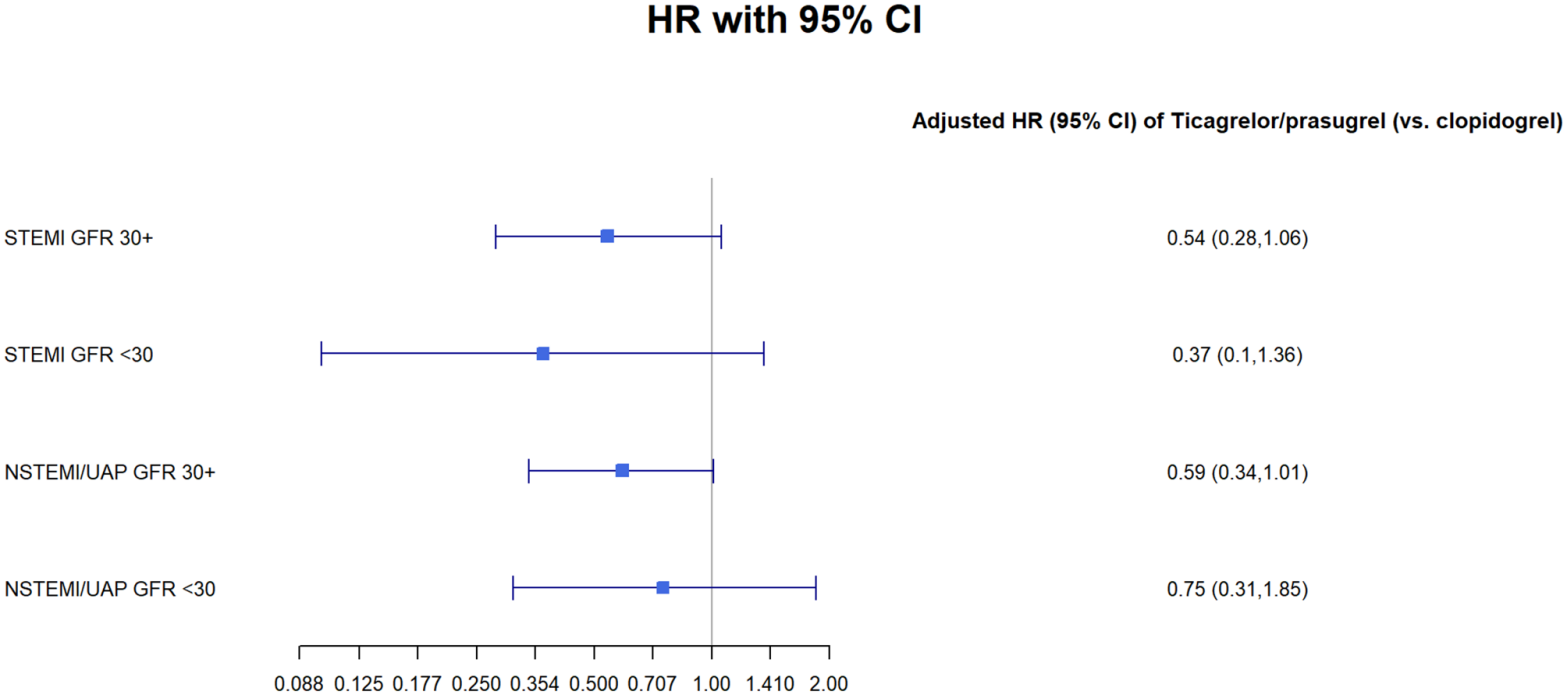
GFR- glomerular filtration rate; STEMI- ST elevation myocardial infraction; NSTEMI- non-ST elevation myocardial infraction; UAP- unstable angina pectoris

We have perform sensitivity analysis using Cox regression model for 1 year mortality for the subpopulation groups of: male, female and patients under and over 65 (Tables 1, 2, 3 and 4 Figs. 1, 2, 3 and 4).

**Table 1:** Cox regression models for 1-year mortality: age < 65

	STEMI GFR 30 +	STEMI GFR < 30	NSTEMI/UAP GFR 30 +	NSTEMI/UAP GFR < 30
Ticagrelor/prasugrel (vs. clopidogrel)	0.32* (0.11, 0.89)	0.88 (0.08, 9.78)	0.79 (0.27, 2.27)	0.48 (0.05, 4.73)
p-value	0.03	0.92	0.67	0.54
Male	0.22** (0.08, 0.60)		2.42 (0.32, 18.56)	0.19 (0.03, 1.40)
p-value	0.003		0.40	0.11
EF < 40%	2.50 (0.98, 6.37)	2.38 (0.21, 26.58)	1.23 (0.27, 5.48)	2.90 (0.40, 20.87)
p-value	0.06	0.49	0.80	0.30
Observations	1,337	23	1,381	45

Note *p < 0.05, **p < 0.01

**Table 2:** Cox regression models for 1-year mortality: age 65 +

Model	Ticagrelor/Prasugrel vs. Clopidogrel	p-value	Male	p-value	EF < 40%	p-value	Observations
STEMI GFR ≥ 30	0.46** (0.25, 0.83)	0.01	0.77 (0.41, 1.43)	0.41	2.14* (1.18, 3.86)	0.02	770
STEMI GFR < 30	0.21* (0.05, 0.77)	0.02	0.73 (0.25, 2.11)	0.57	2.85 (0.93, 8.73)	0.07	55
NSTEMI/UAP GFR ≥ 30	0.51** (0.31, 0.82)	0.01	0.77 (0.48, 1.22)	0.27	2.79*** (1.79, 4.36)	0.0000	1,365
NSTEMI/UAP GFR < 30	0.65 (0.29, 1.49)	0.32	1.34 (0.65, 2.75)	0.43	1.99* (1.05, 3.76)	0.04	151

Note *p < 0.05, **p < 0.01, ***p < 0.001

**Table 3:** Cox regression models for 1-year mortality: Females

Model	STEMI GFR ≥ 30	STEMI GFR < 30	NSTEMI/UAP GFR ≥ 30	NSTEMI/UAP GFR < 30
Ticagrelor/prasugrel (vs. clopidogrel)	0.67 (0.25, 1.79)	0.17 (0.02, 1.46)	1.29 (0.58, 2.86)	0.45 (0.10, 2.06)
p-value	0.43	0.11	0.54	0.31
Age (per year)	1.04* (1.00, 1.09)	1.00 (0.94, 1.07)	1.11*** (1.06, 1.16)	1.02 (0.94, 1.09)
p-value	0.05	0.93	0.0000	0.68
EF < 40%	1.56 (0.64, 3.80)	3.26 (0.62, 17.18)	1.91 (0.87, 4.18)	1.48 (0.43, 5.06)
p-value	0.33	0.17	0.11	0.54
Observations	342	26	601	55

Note *p < 0.05, **p < 0.01, ***p < 0.001

**Table 4:** Cox regression models for 1-year mortality: Males

	STEMI GFR 30 +	STEMI GFR < 30	NSTEMI/UAP GFR 30 +	NSTEMI/UAP GFR < 30
Ticagrelor/prasugrel (vs. clopidogrel)	0.54	0.37	0.59	0.75
	(0.28, 1.06)	(0.10, 1.36)	(0.34, 1.01)	(0.31, 1.85)
p-value	0.08	0.14	0.06	0.54
Age (per year)	1.07***	1.01	1.08***	1.06**
	(1.04, 1.10)	(0.96, 1.05)	(1.05, 1.10)	(1.02, 1.10)
p-value	0.0000	0.80	0.00	0.003
EF < 40%	2.38**	2.02	2.42***	2.16*
	(1.29, 4.37)	(0.55, 7.35)	(1.46, 4.02)	(1.06, 4.40)
p-value	0.01	0.29	0.001	0.04
Observations	1,765	52	2,145	141
Note:	*p < 0.05	**p < 0.01	***p < 0.001	

## Results

**Fig. 5:**
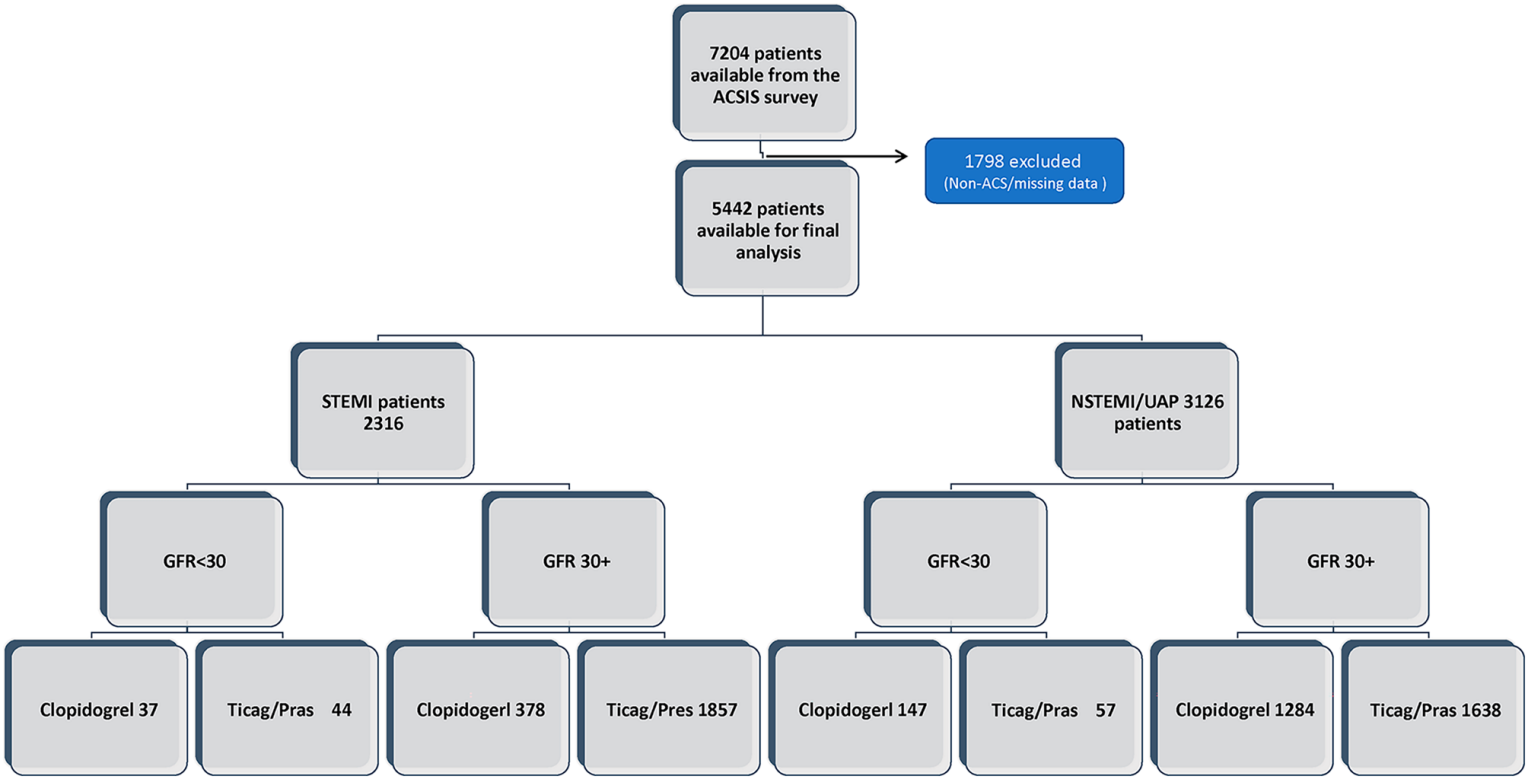
ACSIS-ACS Israeli Surveys; GFR- glomerular filtration rate; STEMI- ST elevation myocardial infraction; NSTEMI- non-ST elevation myocardial infraction; UAP- unstable angina pectoris; Clop–Clopidogrel; Ticag/Pras- Ticagrelor /Prasugrel

A total of 7,204 patients were initially analyzed with 1,762 excluded due to insufficient data or in-hospital mortality, with a final total of 5442 patients included in the study. Of them 2316 (42.5%) patients were diagnosed with STEMI, and 3126 (57.5%) were diagnosed with NSTEMI/UAP (Fig. 5).

### Mortality and outcomes

In low GFR patients, both with STEMI and NSTEM/UAP, there were no differences in 30-day outcomes for rehospitalization, recurrent MI, and major or minor bleeding 10 between clopidogrel or ticagrelor/prasugrel antiplatelet therapy (for the STEMI patients: 32.3% vs. 29.4% p = 1.0, 3.2% vs. 2.8, p = 1.0, 3.2% vs. 0 %, p = 0.94, and 0% vs. 2.8%, p = 1.0, respectively and for the NSTEMI/UAP 20% vs 8% p = 0.085,0%vs 0% p = not relevant, 0% vs 1.6% p = 0.9, 0% vs 0 % p = not relevant, respectively). Additionally, there was no difference in the combined outcome of ischemia and minor/major bleeding (for STEMI patients:6.5% vs.11.1%,p = 0.81 and for NSTEMI/UAP patients:7.3% vs 6% p = 1.0) (Table 5–6). Importantly, the starting point was at hospital admission.

**Table 5:** Outcome by diagnosis and glomerular filtration rate

	STEMI				NSTEMI/UAP			
	Overall	GFR < 30	GFR 30 +	p	Overall	GFR < 30	GFR 30 +	p
n	2316	81	2235		3126	204	2922	
**Outcomes in 30-days from admission (not including in-hospital events)**								
Re-hospitalization	307 (15.5)	20 (30.8)	287 (15.0)	0.001	383 (14.6)	29 (16.7)	354 (14.4)	0.483
RE–MI	33 (1.7)	2 (3.0)	31 (1.6)	0.718	43 (1.7)	0 (0.0)	43 (1.8)	0.140
Major bleeding	3 (0.2)	1 (1.5)	2 (0.1)	0.207	9 (0.4)	2 (1.2)	7 (0.3)	0.233
Minor bleeding	13 (0.7)	1 (1.5)	12 (0.6)	0.933	17 (0.7)	0 (0.0)	17 (0.7)	0.531
PCI	141 (7.2)	3 (4.5)	138 (7.3)	0.527	119 (4.7)	5 (2.9)	114 (4.8)	0.348
CABG	32 (1.6)	0 (0.0)	32 (1.7)	0.560	81 (3.2)	5 (2.9)	76 (3.2)	1.000
Combined outcome (–, PCI, CABG, minor/major bleeding)	204 (10.4)	6 (9.0)	198 (10.4)	0.856	238 (9.3)	12 (6.9)	226 (9.5)	0.334
Combined outcome (–, PCI, CABG)	188 (9.6)	4 (6.0)	184 (9.7)	0.422	218 (8.5)	10 (5.8)	208 (8.7)	0.235
Outcomes ischemic vs. bleeding:				NaN				0.518
REMI/PCI/CABG	188 (9.6)	4 (6.0)	184 (9.7)		212 (8.3)	10 (5.8)	202 (8.4)	
Bleeding (major/minor)	16 (0.8)	2 (3.0)	14 (0.7)		20 (0.8)	2 (1.2)	18 (0.8)	
Both	0 (0.0)	0 (0.0)	0 (0.0)		6 (0.2)	0 (0.0)	6 (0.3)	
No event	1764 (89.6)	61 (91.0)	1703 (89.6)		2326 (90.7)	161 (93.1)	2165 (90.5)	
**Death rates**								
30-day mortality	20 (0.9)	4 (5.0)	16 (0.7)	0.001	16 (0.5)	2 (1.0)	14 (0.5)	0.652
1-year mortality	80 (3.7)	17 (21.8)	63 (3.0)	<0.001	137 (4.7)	43 (21.9)	94 (3.4)	<0.001

**Table 6:** Outcome by Medication

	STEMI						NSTEMI/UAP					
	GFR < 30			GFR 30 +			GFR < 30			GFR 30 +		
	Clop	Ticag/Pras	p	Clop	Ticag/Pras	p	Clop	Ticag/Pras	p	Clop	Ticag/Pras	p
n	37	44		378	1857		147	57		1284	1638	
**Outcomes in 30-days from admission (not including in-hospital events)**												
Re-hospitalization	10 (32.3)	10 (29.4)	1.000	60 (19.1)	227 (14.1)	0.030	25 (20.2)	4 (8.0)	0.085	161 (15.4)	193 (13.7)	0.276
RE–MI	1 (3.2)	1 (2.8)	1.000	4 (1.3)	27 (1.7)	0.763	0 (0.0)	0 (0.0)	NaN	20 (1.9)	23 (1.7)	0.762
Major bleeding	1 (3.2)	0 (0.0)	0.940	2 (0.6)	0 (0.0)	0.026	2 (1.6)	0 (0.0)	0.912	4 (0.4)	3 (0.2)	0.711
Minor bleeding	0 (0.0)	1 (2.8)	1.000	4 (1.3)	8 (0.5)	0.241	0 (0.0)	0 (0.0)	NaN	9 (0.9)	8 (0.6)	0.562
PCI	1 (3.2)	2 (5.6)	1.000	15 (4.8)	123 (7.8)	0.082	4 (3.3)	1 (2.0)	1.000	39 (3.8)	75 (5.5)	0.061
CABG	0 (0.0)	0 (0.0)	NaN	10 (3.2)	22 (1.4)	0.042	3 (2.4)	2 (4.1)	0.939	52 (5.1)	24 (1.8)	<0.001
Combined outcome (RE–MI, PCI, CABG, minor/major bleeding)	2 (6.5)	4 (11.1)	0.813	35 (11.1)	163 (10.3)	0.717	9 (7.3)	3 (6.0)	1.000	110 (10.7)	116 (8.5)	0.084
Combined outcome (RE–MI, PCI, CABG)	1 (3.2)	3 (8.3)	0.717	29 (9.2)	155 (9.8)	0.852	7 (5.7)	3 (6.0)	1.000	102 (9.9)	106 (7.8)	0.079
Outcomes ischemic vs. bleeding:			NaN			NaN			NaN			0.096
REMI/PCI/CABG	1 (3.2)	3 (8.3)		29 (9.2)	155 (9.8)		7 (5.7)	3 (6.0)		97 (9.4)	105 (7.7)	
Bleeding (major/ minor)	1 (3.2)	1 (2.8)		6 (1.9)	8 (0.5)		2 (1.6)	0 (0.0)		8 (0.8)	10 (0.7)	
Both	0 (0.0)	0 (0.0)		0 (0.0)	0 (0.0)		0 (0.0)	0 (0.0)		5 (0.5)	1 (0.1)	
No event	29 (93.5)	32 (88.9)		279 (88.9)	1424 (89.7)		114 (92.7)	47 (94.0)		919 (89.3)	1246 (91.5)	
**Death rates**												
30-day mortality	3 (8.1)	1 (2.3)	0.504	7 (1.9)	9 (0.5)	0.011	1 (0.7)	1 (1.8)	1.000	10 (0.8)	4 (0.2)	0.072
1-year mortality	12 (33.3)	5 (11.9)	0.044	27 (7.6)	36 (2.1)	<0.001	35 (24.5)	8 (15.1)	0.224	62 (5.0)	32 (2.1)	<0.001

**Fig. 6:**
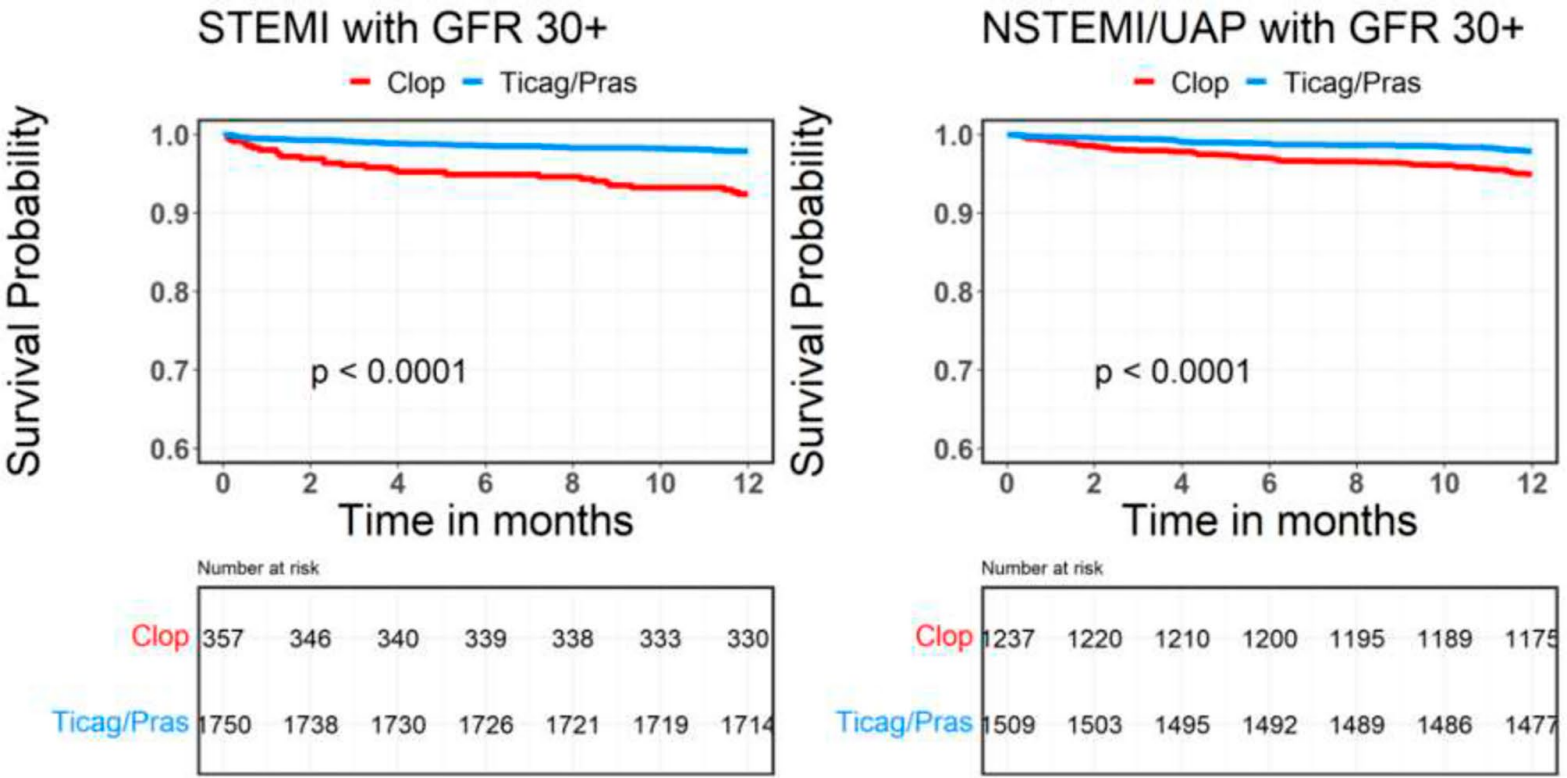
GFR- glomerular filtration rate; STEMI- ST elevation myocardial infraction; NSTEMI- non-ST elevation myocardial infraction; Clop–Clopidogrel; Ticag/Pras- Ticagrelor/Prasugrel

**Fig. 7:**
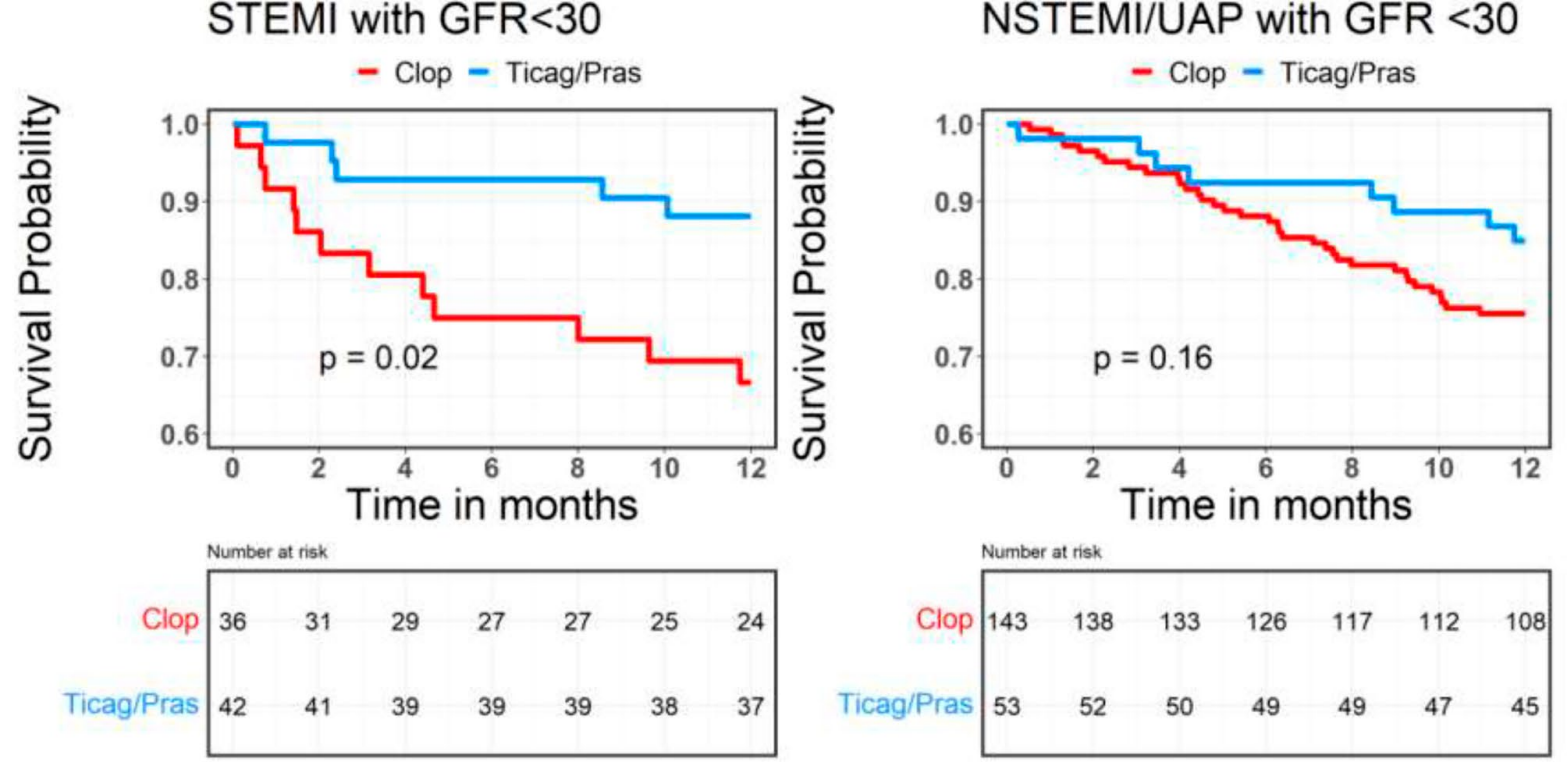
GFR- glomerular filtration rate; STEMI- ST elevation myocardial infraction; NSTEMI- non-ST elevation myocardial infraction; Clop–Clopidogrel; Ticag/Pras- Ticagrelor /Prasugrel

Thirty day and 1-year mortality rates were lower in patients with higher GFR regardless of the type of ACS. In the lower GFR group, there was no significant difference in the 30-day mortality rate between the two different regimens in patients with either STEMI or NSTEMI/UAP. However, there was a lower mortality rate at 1 year in CKD-STEMI patients treated with ticagrelor/prasugrel as compared with clopidogrel (33.3% VS. 11.9%, P = 0.04). In patients with NSTEMI/UAP and low GFR there was no significant difference between the two treatment regimens (Fig. 6 and 7).

In a multivariate analysis including age, sex, and EF, there was no significant different in 30-days outcomes including rehospitalization, recurrent MI, and major or minor bleeding for all groups, however,there was a significance difference in 1-year mortality rate in the STEMI-CKD group treated with Ticagrelor/Prasugrel (HR = 0.28 CI 0.1–0.83 p < 0.05)

Propensity score analysis: To reduce bias between the groups of GFR, a propensity score matching (PSM) was performed, in the STEMI patients and in the NSTEMI/UAP patients. There was no significant change in the outcomes parameters at 30 days all groups. There was a significant difference in the mortality rate in year in the STEMI group with low GFR.

In a sensitivity analysis we found a statistically significant difference in patients treated with the Ticagrelor/prasugrel in both the under and over 65 groups (p < 0.05). We didn’t find any difference in the sub analysis for either male of female (>0.05).

### Patients characteristics

#### STEMI patients

Among the STEMI group, patients with CKD were older and had a higher rate of female gender (73 years vs 60, p < 0.001, and 33.3% vs 16.6%, p < 0.001 respectively). They had a higher prevalence of major cardiovascular risk factors and cardiovascular comorbidities as shown in Table 7. Interestingly they were more often under previous antiplatelet therapy with either aspirin or clopidogrel at the time of presentation (57% vs. 31%, p < 0.001, 15.5 % vs. 5.2%, p < 0.001, respectively). CKD-STEMI patients had higher rates of three vessel coronary artery disease (33.8% vs. 20.1% p < 0.007) and cardiogenic shock (19.8% vs. 1.6%, p < 0.001) as compared with non-CKD STEMI patients as shown in (Table 7).

**Table 7:** Baseline characteristic according to diagnosis and GFR

	STEMI				NSTEMI/UAP			
	Overall	GFR < 30	GFR 30 +	p	Overall	GFR < 30	GFR 30 +	p
n	2316	81	2235		3126	204	2922	
**Baseline characteristics**								
Age, years (median [IQR])	60.00 [52.00, 69.00]	73.00 [63.00, 83.00]	60.00 [52.00, 69.00]	<0.001	65.00 [57.00, 73.00]	72.00 [65.00, 80.00]	64.00 [56.00, 73.00]	<0.001
Gender (male)	1919 (82.9)	54 (66.7)	1865 (83.4)	<0.001	2436 (77.9)	146 (71.6)	2290 (78.4)	0.029
Dyslipidemia	1495 (64.8)	57 (70.4)	1438 (64.6)	0.342	2416 (77.4)	167 (81.9)	2249 (77.1)	0.137
Hypertension	1209 (52.3)	65 (80.2)	1144 (51.3)	<0.001	2234 (71.6)	192 (94.1)	2042 (70.0)	<0.001
Current smokers	1185 (51.2)	19 (23.5)	1166 (52.2)	<0.001	1160 (37.1)	49 (24.0)	1111 (38.0)	<0.001
Diabetes mellitus	752 (32.5)	43 (53.1)	709 (31.8)	<0.001	1424 (45.6)	148 (72.9)	1276 (43.7)	<0.001
Family history of CAD	666 (32.5)	11 (16.9)	655 (33.0)	0.010	827 (31.5)	32 (20.5)	795 (32.2)	0.003
BMI (kg/m2), (mean ± SD)	27.04 [24.51, 30.04]	26.62 [24.28, 29.48]	27.07 [24.51, 30.04]	0.475	27.68 [24.86, 30.88]	27.72 [24.64, 32.84]	27.68 [24.90, 30.86]	0.280
Prior MI	536 (23.2)	26 (32.5)	510 (22.8)	0.060	1380 (44.3)	131 (64.5)	1249 (42.9)	<0.001
Prior CABG	63 (2.7)	4 (4.9)	59 (2.6)	0.367	362 (11.6)	40 (19.6)	322 (11.0)	<0.001
Prior PCI	521 (22.5)	23 (28.4)	498 (22.3)	0.247	1326 (42.5)	117 (57.6)	1209 (41.5)	<0.001
PVD	87 (3.8)	7 (8.6)	80 (3.6)	0.040	254 (8.1)	50 (24.5)	204 (7.0)	<0.001
s/p CVA/TIA	139 (6.0)	10 (12.3)	129 (5.8)	0.028	289 (9.2)	43 (21.1)	246 (8.4)	<0.001
History of CHF	68 (2.9)	12 (14.8)	56 (2.5)	<0.001	298 (9.5)	74 (36.6)	224 (7.7)	<0.001
Grace score > 140	89 (4.2)	19 (25.3)	70 (3.4)	<0.001	467 (15.8)	111 (58.4)	356 (12.9)	<0.001
EF class (%):				<0.001				<0.001
Normal (EF > 50%)	769 (37.8)	12 (17.9)	757 (38.5)		1532 (62.7)	79 (47.3)	1453 (63.8)	
Mild (EF 40–50%)	748 (36.8)	19 (28.4)	729 (37.1)		542 (22.2)	36 (21.6)	506 (22.2)	
Moderate (EF 30–40%)	418 (20.6)	22 (32.8)	396 (20.2)		277 (11.3)	39 (23.4)	238 (10.5)	
Severe (EF < 30%)	97 (4.8)	14 (20.9)	83 (4.2)		92 (3.8)	13 (7.8)	79 (3.5)	
Number of vessels diseased:				0.007				<0.001
None	118 (5.5)	7 (9.9)	111 (5.3)		93 (4.4)	7 (6.4)	86 (4.3)	
1 vessel	912 (42.3)	23 (32.4)	889 (42.6)		660 (31.3)	17 (15.6)	643 (32.2)	
2 vessels	685 (31.7)	17 (23.9)	668 (32.0)		708 (33.6)	29 (26.6)	679 (34.0)	
3 vessels	443 (20.5)	24 (33.8)	419 (20.1)		645 (30.6)	56 (51.4)	589 (29.5)	
Hospital duration in days (median [IQR])	4.00 [3.00, 5.00]	6.00 [4.00, 8.00]	4.00 [3.00, 5.00]	<0.001	3.00 [2.00, 5.00]	6.00 [4.00, 9.75]	3.00 [2.00, 5.00]	<0.001

#### NSTEMI/UAP patients

NSTEMI/UAP-CKD patients were older with higher prevalence of cardiovascular comorbidities as shown in Table 7. They also suffered more from three vessel CAD (51.4% vs 29.5% p < 0.001) and from cardiogenic shock (2.9% vs. 0.4% p < 0.001), when compared to non-CKD NSTEMI patients.

#### Antiplatelet treatment

Among CKD-STEMI patients, patients treated with Ticagrelor/Prasugrel were younger than those treated with vs. Clopidogrel. CKD-NSTEMI patients treated with ticagrelor/prasugrel were younger than the NSTEMI patients treated with clopidogrel but had similar comorbidities, extent of CAD, and in-hospital complications (Table 8).

**Table 8:** Baseline characteristic by medication

	STEMI						NSTEMI/UAP					
	GFR < 30			GFR 30 +			GFR < 30			GFR 30 +		
	Clop	Ticag/ Pras	p	Clop	Ticag/ Pras	p	Clop	Ticag/ Pras	p	Clop	Ticag/ Pras	p
n	37	44		378	1857		147	57		1284	1638	
**Baseline characteristics**												
Age, years (median [IQR])	78.00 [68.00, 85.00]	69.00 [59.00, 78.50]	0.011	71.00 [58.25, 80.00]	59.00 [51.00, 66.00]	<0.001	74.00 [67.00, 80.50]	69.00 [62.00, 78.00]	0.049	67.00 [58.00, 77.00]	62.00 [54.00, 70.00]	<0.001
Gender (male)	21 (56.8)	33 (75.0)	0.134	284 (75.1)	1581 (85.1)	<0.001	106 (72.1)	40 (70.2)	0.919	954 (74.3)	1336 (81.6)	<0.001
Dyslipidemia	25 (67.6)	32 (72.7)	0.793	248 (66.3)	1190 (64.3)	0.485	120 (81.6)	47 (82.5)	1.000	1037 (80.9)	1212 (74.1)	<0.001
Hypertension	30 (81.1)	35 (79.5)	1.000	245 (65.3)	899 (48.5)	<0.001	141 (95.9)	51 (89.5)	0.155	954 (74.4)	1088 (66.5)	<0.001
Current smokers	6 (16.2)	13 (29.5)	0.251	134 (35.4)	1032 (55.6)	<0.001	41 (27.9)	8 (14.0)	0.058	413 (32.2)	698 (42.6)	<0.001
Diabetes mellitus	21 (56.8)	22 (50.0)	0.701	132 (35.1)	577 (31.1)	0.145	109 (74.7)	39 (68.4)	0.470	595 (46.4)	681 (41.7)	0.012
Family history of CAD	1 (3.6)	10 (27.0)	0.031	64 (19.5)	591 (35.6)	<0.001	24 (21.2)	8 (18.6)	0.887	288 (26.8)	507 (36.4)	<0.001
BMI (kg/m2), (mean ± SD)	26.61 [24.08, 29.33]	26.81 [24.43, 29.92]	0.735	26.26 [23.88, 29.70]	27.29 [24.69, 30.08]	0.003	27.41 [24.63, 32.87]	28.48 [24.80, 31.96]	0.547	27.69 [24.97, 30.85]	27.55 [24.82, 30.86]	0.700
Prior MI	13 (35.1)	13 (30.2)	0.820	97 (25.7)	413 (22.3)	0.170	100 (68.5)	31 (54.4)	0.085	602 (47.0)	647 (39.6)	<0.001
Prior CABG	1 (2.7)	3 (6.8)	0.736	17 (4.5)	42 (2.3)	0.022	30 (20.4)	10 (17.5)	0.790	180 (14.0)	142 (8.7)	<0.001
Prior PCI	11 (29.7)	12 (27.3)	1.000	87 (23.0)	411 (22.1)	0.762	87 (59.6)	30 (52.6)	0.457	610 (47.6)	599 (36.7)	<0.001
PVD	2 (5.4)	5 (11.4)	0.580	29 (7.7)	51 (2.7)	<0.001	39 (26.5)	11 (19.3)	0.370	107 (8.3)	97 (5.9)	0.013
s/p CVA/TIA	4 (10.8)	6 (13.6)	0.963	49 (13.0)	80 (4.3)	<0.001	35 (23.8)	8 (14.0)	0.179	145 (11.3)	101 (6.2)	<0.001
History of CHF	6 (16.2)	6 (13.6)	0.991	18 (4.8)	38 (2.0)	0.004	55 (37.7)	19 (33.9)	0.741	126 (9.8)	98 (6.0)	<0.001
Grace score > 140	15 (44.1)	4 (9.8)	0.002	43 (12.3)	27 (1.6)	<0.001	86 (62.3)	25 (48.1)	0.107	239 (19.8)	117 (7.6)	<0.001
EF class (%):			0.739			<0.001			0.929			0.010
Normal (EF > 50%)	6 (20.0)	6 (16.2)		108 (31.5)	649 (40.0)		57 (47.1)	22 (47.8)		618 (63.8)	835 (63.8)	
Mild (EF 40–50%)	10 (33.3)	9 (24.3)		123 (35.9)	606 (37.4)		27 (22.3)	9 (19.6)		193 (19.9)	313 (23.9)	
Moderate (EF 30–40%)	8 (26.7)	14 (37.8)		87 (25.4)	309 (19.1)		27 (22.3)	12 (26.1)		114 (11.8)	124 (9.5)	
Severe (EF < 30%)	6 (20.0)	8 (21.6)		25 (7.3)	58 (3.6)		10 (8.3)	3 (6.5)		43 (4.4)	36 (2.8)	
Number of vessels diseased:			0.424			0.003			0.406			<0.001
None	4 (12.9)	3 (7.5)		18 (5.2)	93 (5.3)		4 (5.4)	3 (8.6)		61 (6.7)	25 (2.3)	
1 vessel	7 (22.6)	16 (40.0)		126 (36.4)	763 (43.8)		12 (16.2)	5 (14.3)		255 (27.9)	388 (35.9)	
2 vessels	9 (29.0)	8 (20.0)		108 (31.2)	560 (32.2)		23 (31.1)	6 (17.1)		296 (32.3)	383 (35.4)	
3 vessels	11 (35.5)	13 (32.5)		94 (27.2)	325 (18.7)		35 (47.3)	21 (60.0)		303 (33.1)	286 (26.4)	
Hospital Duration In days (median [IQR])	6.00 [4.00, 8.00]	5.00 [3.00, 7.50]	0.269	4.00 [3.00, 7.00]	4.00 [3.00, 5.00]	<0.001	5.50 [4.00, 9.00]	6.00 [4.00, 10.00]	0.679	4.00 [2.00, 5.00]	3.00 [2.00, 4.00]	<0.001

#### Antiplatelet treatment and GFR levels

**Fig. 8:**
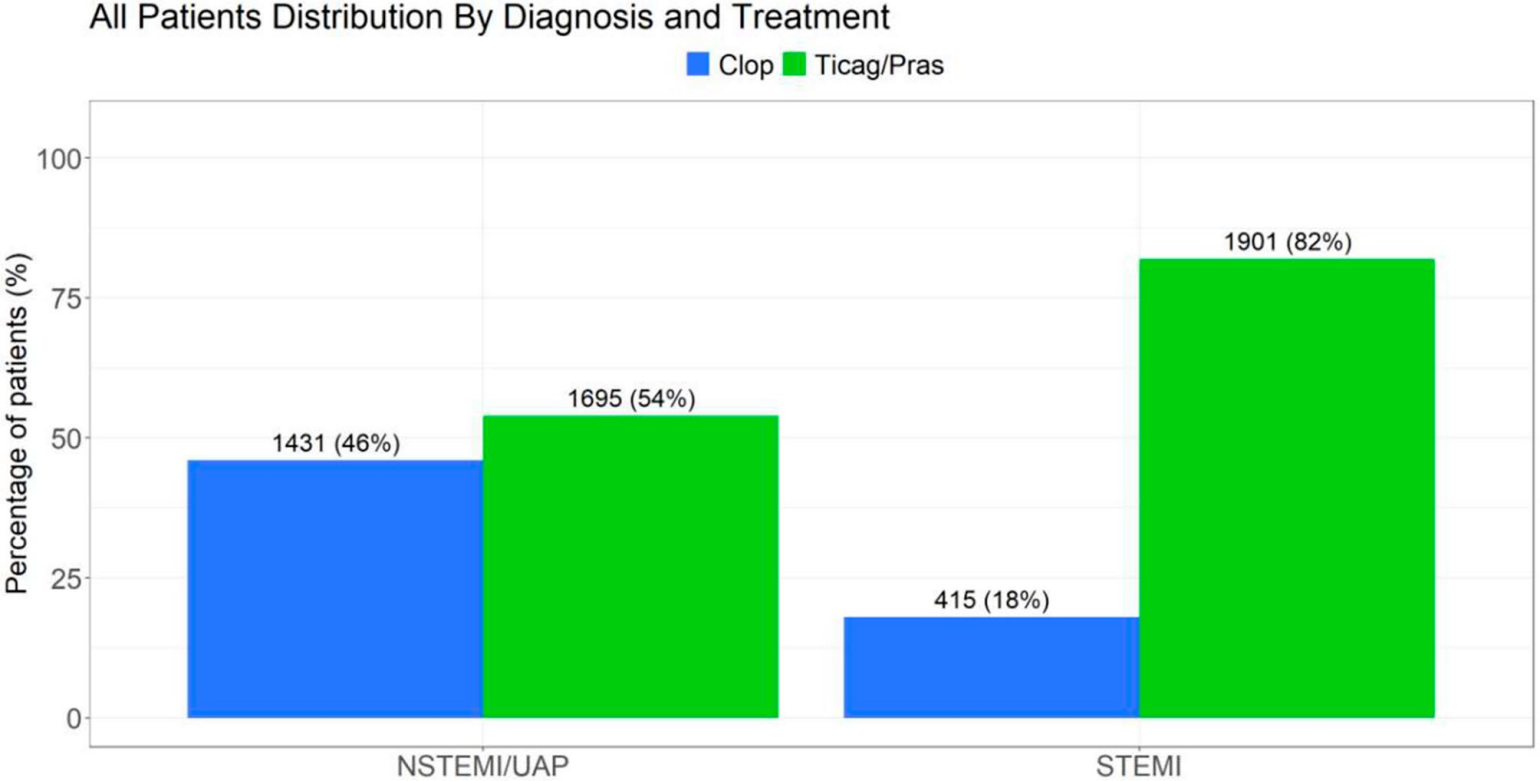
STEMI- ST elevation myocardial infraction; NSTEMI- non-ST elevation myocardial infraction

**Fig. 9:**
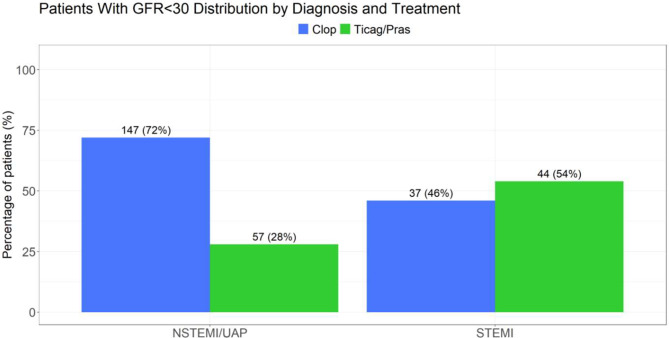
GFR- glomerular filtration rate; STEMI- ST elevation myocardial infraction; NSTEMI- non-ST elevation myocardial infraction

**Fig. 10:**
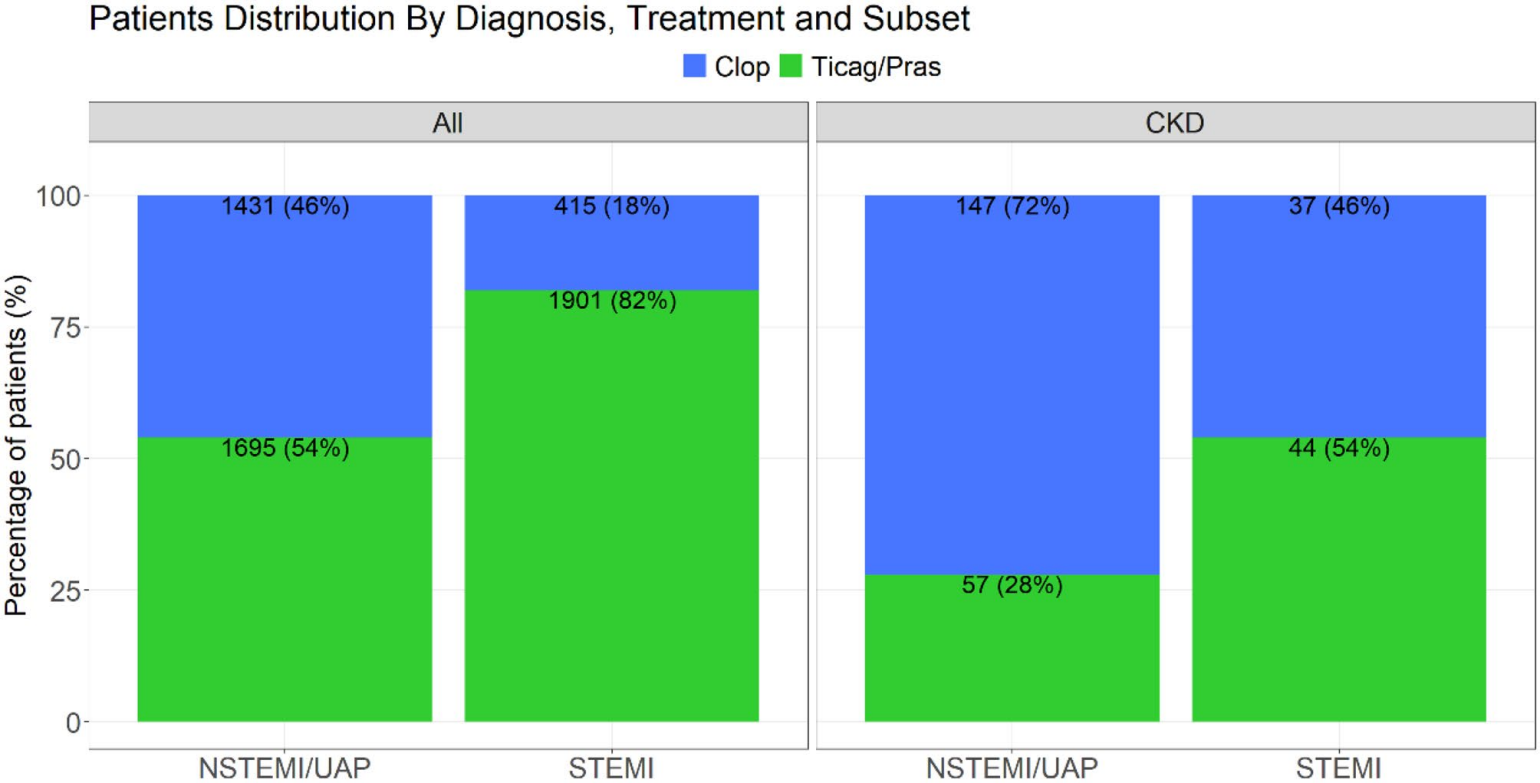
GFR- glomerular filtration rate; STEMI- ST elevation myocardial infraction; NSTEMI- non-ST elevation myocardial infraction

Low level GFR (<30 ml/min) was found in 81/2316 (3.5%) patients presenting with STEMI and 204/3126 (6.5%) in the NSTEMI/UAP patients. Of the CKD-STEMI patients, 37/81 (45.6%) received clopidogrel and 44/81 (54.4%) received ticagrelor/prasugrel. Of the CKD-NSTEMI/UAP patients 147/204 (72%) received clopidogrel while 57/204 (28%) patients received ticagrelor/prasugrel (Figure 8, 9 and 10).

## Discussion

The main findings of our study were: a) patients with CKD were sicker and had a higher mortality irrespective of the type of ACS; b) there was no difference in 30-day outcomes in patients with GFR < 30 ml/min regarding their antiplatelet regimen and c) at 1-year, CKD-STEMI patients treated with ticagrelor/prasugrel had a lower mortality rate when compared to clopidogrel treatment.

The baseline characteristics of the CKD patients and their mortality rates in our study are consistent with previous findings showing that these patients have higher mortality and are treated with less aggressive antiplatelet regimens [15–17].

The similarity of 30-day outcomes in patients with GFR < 30 ml/min might be explained, by the relatively small number of these patients in our study, and/or due the early phase after ACS. The lower 1-year mortality seen with ticagrelor/prasugrel treatment in STEMI patients, both in the unadjusted and the adjusted analyses, is consistent with previous studies [18].

Several prior studies have compared ticagrelor/prasugrel to clopidogrel in patients with impaired renal function. These studies showed that the improvements in mortality driven by the reduction of ischemic events with ticagrelor/prasugrel was often offset by an increased bleeding risk [7, 19].

In our study, there was mortality benefit for the potent antiplatelets regimen in patients with GFR < 30 after STEMI without an increase of bleeding events at 30-day follow up. This might be explained by the younger age of the STEMI patients treated with Ticagrelor/Prasugrel vs. Clopidogrel group (69 y.o vs. 78 y.o, respectively). As shown previously, STEMI patients are usually younger than NSTEMI patients and often have lower bleeding rates [20–22].

Additionally, major bleeding after ACS occurring early after dual antiplatelet therapy (DAPT) initiation are associated with a higher mortality [23]. Hence, our finding showing no difference in the bleeding rate at 30-day, an important prognostic time frame, might shed more light on the constant dilemma between the balance of a high bleeding risk versus a high ischemic risk.

High potency antiplatelet therapy with ticagrelor/prasugrel is a well established treatment choice for ACS patients, especially in patients with higher thrombotic risk [5, 6]. In patients with CKD with lower GFR, bleeding risk may limit use of more potent antiplatelet regimens [24]. Most previous trials examining CKD population used a GFR cutoff of < 60 ml/min, while we investigated patients with more advanced CKD (i.e. GFR < 30 ml/min). Filippo et al showed findings consistent with our study, with lower risk of death and recurrent MI without increased bleeding, but they used a GFR cut-off of 60 ml/min [25]. Roh et al showed superiority of ticagrelor over clopidogrel in patients with GFR between 15–44 ml/min, but most of the patients studied had a GFR in the range of 30–44 ml/min [26]. However, while Edford et al showed benefits of ticagrelor across all strata of GFR, there was no clear reduction of ischemic events with higher rates of bleeding [19]. These finding further demonstrate the difficulty of treating patients with advanced CKD with ACS. Potential mechanisms underlying why prasugrel or ticagrelor might be more favorable than clopidogrel in patients with severe renal dysfunction include the increased prevalence of atherosclerosis, hypercoagulability, and inflammatory state in these patients. Due to these conditions, potent antiplatelet agents, which provide more aggressive inhibition of platelet aggregation, may offer greater clinical benefits. However, it is important to note that patients with severe renal dysfunction are also at higher risk for bleeding, which necessitates a careful balance between the benefits of potent antiplatelet therapy and the associated risks.

Our study, based on a large nationwide cohort across a wide range of years, support the use of a potent antithrombotic regimen in STEMI patients with GFR < 30 ml/min due to the 1-year mortality benefit without excess bleeding.

### Limitation

Our study has several limitations: first, it is a retrospective cohort study. Nevertheless, it is a real-world setting study and include patients from 26 medical centers. Second, the GFR cutoffs (<30 and ≥ 30 ml/min) helped us focus on the severe CKD patients, while patients with mildly impaired renal function were grouped together with patients with good renal function. Lastly, patient follow-up was limited to 1-year. The lack of detailed information regarding dialysis patients. Although patients undergoing dialysis were included in the cohort of those with low glomerular filtration rate (GFR), the study did not specify which patients were on dialysis and which were not. This could impact the interpretation of the findings as dialysis can significantly affect clinical outcomes. Additionally the lack of clarity regarding the exact duration of antiplatelet regimens used in the patient cohort. Additionally, the rates of utilization of other anticoagulation therapies were not consistently documented, which may affect the generalizability of the findings.

## Conclusions

In patients with ACS and CKD, rates of rehospitalization, bleeding, and mortality were similar at 30-day follow-up in patients treated with ticagrelor/prasugrel versus patients treated with clopidogrel. Nevertheless, in patients presenting with STEMI and GFR < 30 ml/min, treatment with ticagrelor/prasugrel was correlated with a lower 1-year mortality as compared with clopidogrel treatment, with no increase in bleeding. These findings suggest benefit of ticagrelor/prasugrel treatment especially for severe CKD patients presenting with STEMI.

## Data Availability

The datasets generated and analyzed during the current study are available in the supplementary file.

## Abbreviation

ODC: Optimal DAPT in CKD
